# Caregiver Stressors Predict Subjective Sleep Quality in African American Family Caregivers

**DOI:** 10.1093/geroni/igaf122.1782

**Published:** 2025-12-31

**Authors:** Miranda McPhillips, Glenna Brewster, Elliane Irani

**Affiliations:** Villanova University, Villanova, Pennsylvania, United States; Emory University, Atlanta, Georgia, United States; Case Western Reserve University, Cleveland, Ohio, United States

## Abstract

There is a known association between caregiving stress and sleep quality in the general caregiving population. This study aims to identify specific caregiving stressors that predict subjective sleep quality among African American family caregivers of adults with chronic or disabling conditions. By focusing on this understudied population, we seek to uncover potential intervention targets to improve their sleep quality and overall health. This is a secondary data analysis of a community-engaged study about the social determinants of sleep health and cardiovascular risk in African American family caregivers. The Pittsburgh Sleep Quality Index was the subjective measure of sleep quality. Four measures of caregiver stress were used: three Caregiver Reaction Assessment subscales 1) lack of family support, 2) financial impact, 3) schedule disruption, and 4) relationship quality with the care recipient, measured by two items from the National Study of Caregiving. Data were analyzed using bivariate analyses and linear regression. Participants (n = 100) were primarily female (85%), caring for a parent (52%) and on average 59 years old. Three quarters of the sample (76%) had poor sleep quality. Lack of family support (r = 0.260), financial impact (r = 0.290), schedule disruption (r = 0.482) and negative relationship (r = 0.339) were positively correlated with sleep quality (p < 0.01). In the multivariate model, after controlling for age, gender and co-residence with the care recipient, schedule disruption (b = 1.651; p = 0.005) and relationship quality (b = 0.552; p = 0.039) remained statistically significant predictors of sleep quality. Tailored interventions aimed at managing caregiving schedules and promoting positive caregiving relationships can improve sleep quality in African American family caregivers.

